# Bilateral Simultaneous Breast Reconstruction with DIEP- and TMG Flaps: Head to Head Comparison, Risk and Complication Analysis

**DOI:** 10.3390/jcm9072031

**Published:** 2020-06-28

**Authors:** Laurenz Weitgasser, Karl Schwaiger, Fabian Medved, Felix Hamler, Gottfried Wechselberger, Thomas Schoeller

**Affiliations:** 1 Department of Plastic and Reconstructive Surgery, Marienhospital Stuttgart, Teaching Hospital of the Eberhard Karls University Tuebingen, Germany, Boeheimstr 37, 70199 Stuttgart, Germany; fabian.medved@gmail.com (F.M.); felix.hamler@vinzenz.de (F.H.); thomas.schoeller@vinzenz.de (T.S.); 2 Department of Plastic and Reconstructive Surgery, Hospital of the Brothers of St. John of God (Barmherzige Brüder), Paracelsus Medical University Salzburg, Austria, Kajetanerpl 1, 5010 Salzburg, Austria; gottfried.wechselberger@bbsalz.at

**Keywords:** free flaps, microsurgical, double free flap, breast reconstruction, microsurgery, DIEP, TMG, TUG, muscle, gracilis, mammary cancer, DCIS

## Abstract

Background: A two center retrospective cohort study of simultaneous bilateral breast reconstructions using double deep inferior epigastric perforator (DIEP) flaps and double transverse myocutaneous/upper gracilis (TMG) flaps was conducted. The aim of this study was to compare surgical procedures, complications, and overall outcome. Patients and Methods: Two study groups, either receiving a simultaneous bilateral breast reconstruction, with double DIEP flaps (*n* = 152) in group 1, or double TMG flaps (*n* = 86) in group 2, were compared. A detailed risk and complication analysis was performed. Patient characteristics, operative time and the need for further operations were evaluated. Results: Double DIEP patients had donor site complications in 23.7% and double TMG patients in 16.3% (*p* = 0.9075, RR 1.45). Flap loss rates of 3.5% (double TMG) and 2.6% (double DIEP) were recorded (*p* = 0.7071, RR 1.33). The need for postoperative lipofilling was significantly higher in double TMG patients (65.1% vs. 38.2 %, *p* = 0.0047, RR 1.71). Conclusion: Complication analysis favors the double DIEP procedure. Donor site morbidity was lower and less severe in the double TMG group. Later fat grafting was more frequently needed after double TMG reconstructions. Further studies, preferably of prospective nature, are needed to evaluate the benefit of bilateral simultaneous breast reconstructions.

## 1. Introduction

With rising numbers of bilateral mastectomies for the treatment of ductal carcinoma in situ (DCIS), and with better understanding of genetic predisposition to breast cancer, as well as significant advancements and aesthetic improvements in postmastectomy reconstruction, the need for bilateral autologous tissue breast reconstruction has increased further in the last decade [1,2,3].

In many cases of unilateral breast cancer or DCIS, particularly in patients with BRCA-1, and BRCA-2 mutations or strong positive cancer family history, a prophylactic bilateral mastectomy is recommended. Other scenarios include bilateral implant failure due to capsular fibrosis or cases of mastectomy due to local tumor relapse after a previous quadrantectomy plus radiotherapy. Microsurgical autologous breast reconstruction became a reliable workhorse in these scenarios and offers substantial and aesthetically natural results [4], as well as high patient satisfaction compared to reconstructions with silicone implants [5,6].

Various free flaps for autologous breast reconstructions are available and now represent routine procedures for breast reconstruction after skin-sparing or conventional mastectomy and breast conserving treatment. The deep inferior epigastric perforator (DIEP) flap became a gold standard [4] for breast reconstruction in patients with sufficient amounts of abdominal donor tissue [7]. In patients who do not offer enough abdominal donor tissue for reconstruction because of low body mass index or where the deep inferior epigastric vessels might have been damaged by prior abdominal surgeries, a vast number of alternative free flaps are available. With its low donor site morbidity and concealed scar, the transverse myocutaneous gracilis (TMG) flap is one of the many favored options for the reconstruction of small to medium sized breasts [8,9].

The opportunity to immediately restore like with like, and therefore, preventing the temporary distress of a mastectomy as well as the possibility to reduce the reconstructive process to as few surgeries as possible, increases the request for immediate reconstructions. For these accountable reasons, patients’ preferences are mainly towards immediate, ideally bilateral, breast reconstruction [5,10,11].

Although bilateral simultaneous breast reconstruction is well adapted, with double DIEP as well as with double TMG flaps [6], long operating time, increased blood loss, and doubled donor sites pose certain drawbacks.

The complexity and the risk profile of bilateral simultaneous microsurgical breast reconstructions with autologous tissue is high. To the authors knowledge, a large sized comparative study of both procedures has not been conducted so far. The authors aim was to directly compare and analyze the two immediate reconstructive methods (double DIEP vs. double TMG) in their patient population in respect to complications, and operative time, and the need for future refinement surgery such as autologous fat grafting.

## 2. Patients and Methods

### 2.1. Data Extraction

A total of 119 patients, receiving 238 free flaps during the period of March 2010 and March 2019, were evaluated in the form of a double-center retrospective cohort study. Patients requiring bilateral breast reconstruction were recruited and treated either at the Department for Hand-, Micro- and Reconstructive Surgery at Marienhospital Stuttgart, Germany or at the Department for Plastic, Reconstructive and Aesthetic Surgery at the Hospital of the Brothers of St. John of God Salzburg, Austria. In total, 76 patients received a simultaneous bilateral breast reconstruction with double DIEP flaps, and 43 patients with double TMG flaps.

Patient characteristics, including age, BMI, comorbidities, form of breast disease, medication, gene mutation, smoking history, radiation therapy, were obtained from medical records. Complications were specifically assessed separately in detail and classified according to the following categories: vascular insufficiency, partial flap necrosis, total flap loss, late fat necrosis, donor site complication and erythrocyte concentrate substitution. Additionally, patients were screened for postoperative need of lipofilling for later breast symmetrization.

All subjects gave their informed consent for inclusion before they participated in the study and the study was conducted in accordance with the Declaration of Helsinki.

### 2.2. Surgical Approach

**Figure 1:**
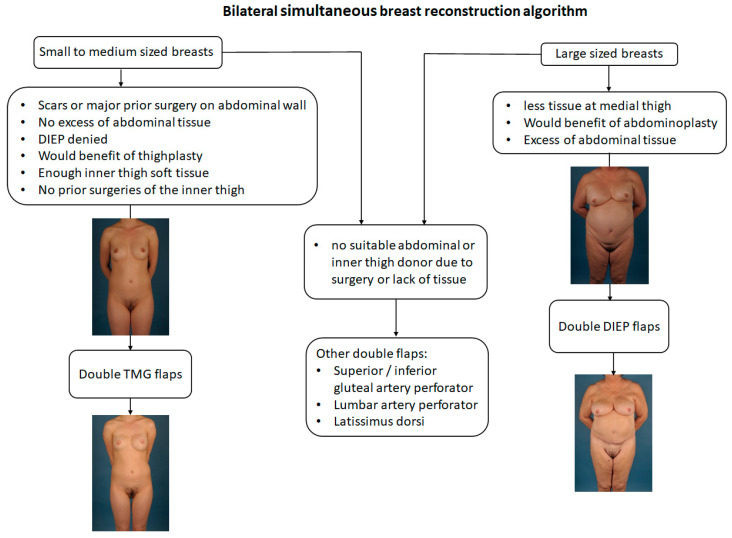
Algorithm for bilateral breast reconstruction with autologous tissue.

**Figure 2:**
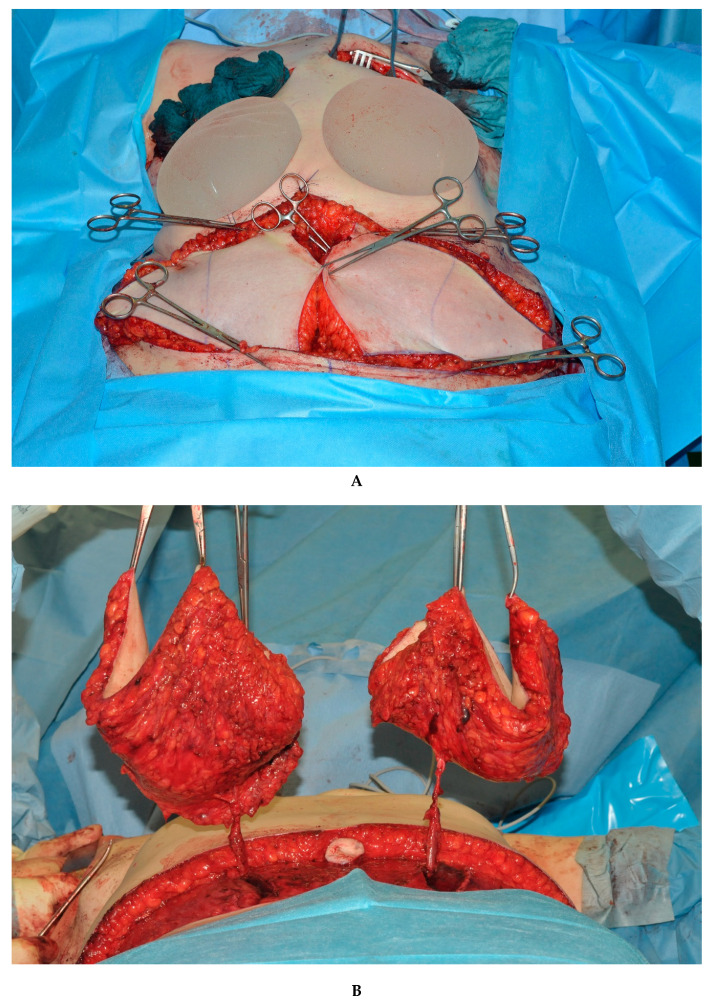

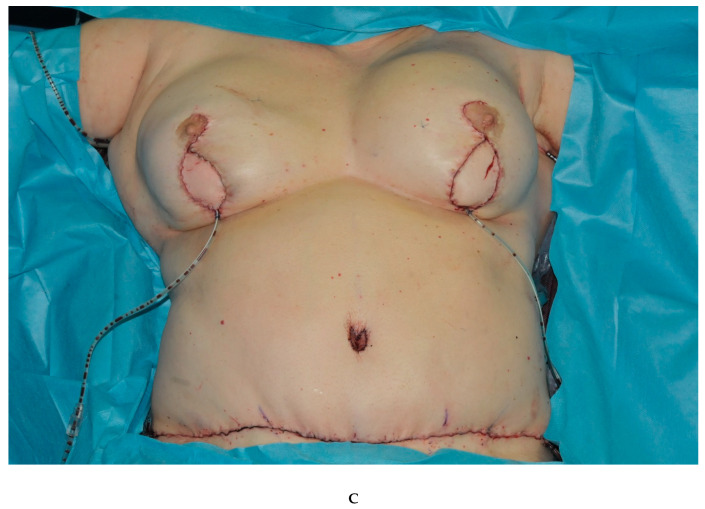
(**A**). Bilateral simultaneous breast reconstruction with DIEP flaps. (**B**). Raised bilateral DIEP flaps. (**C**). Intraoperative result after breast reconstruction with simultaneous bilateral double DIEP flaps.

**Figure 3:**
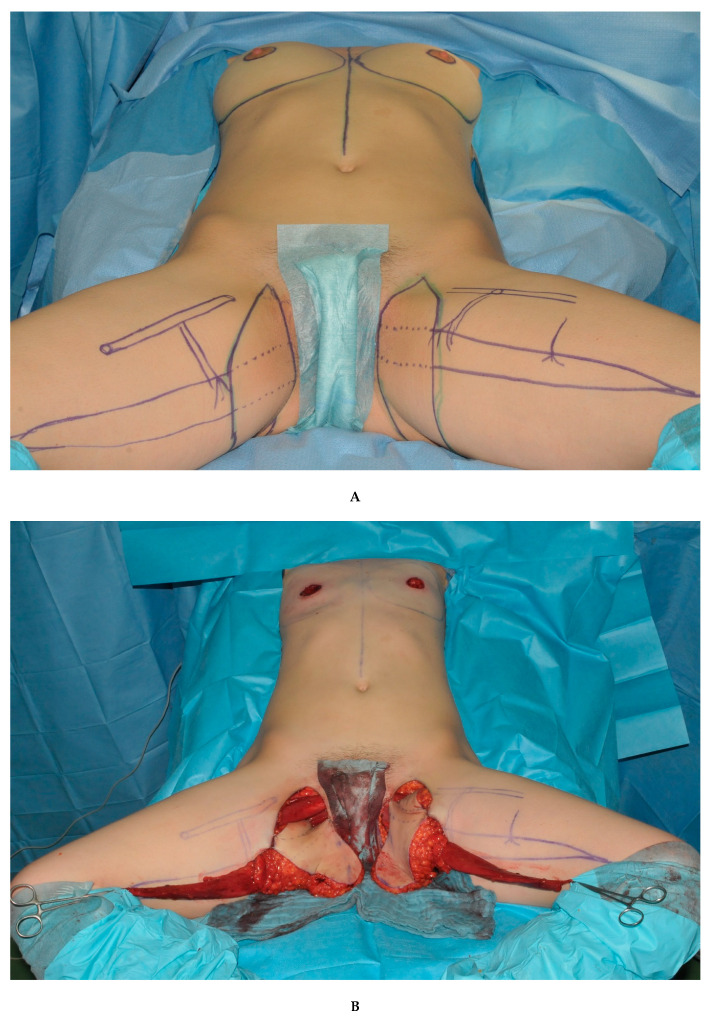

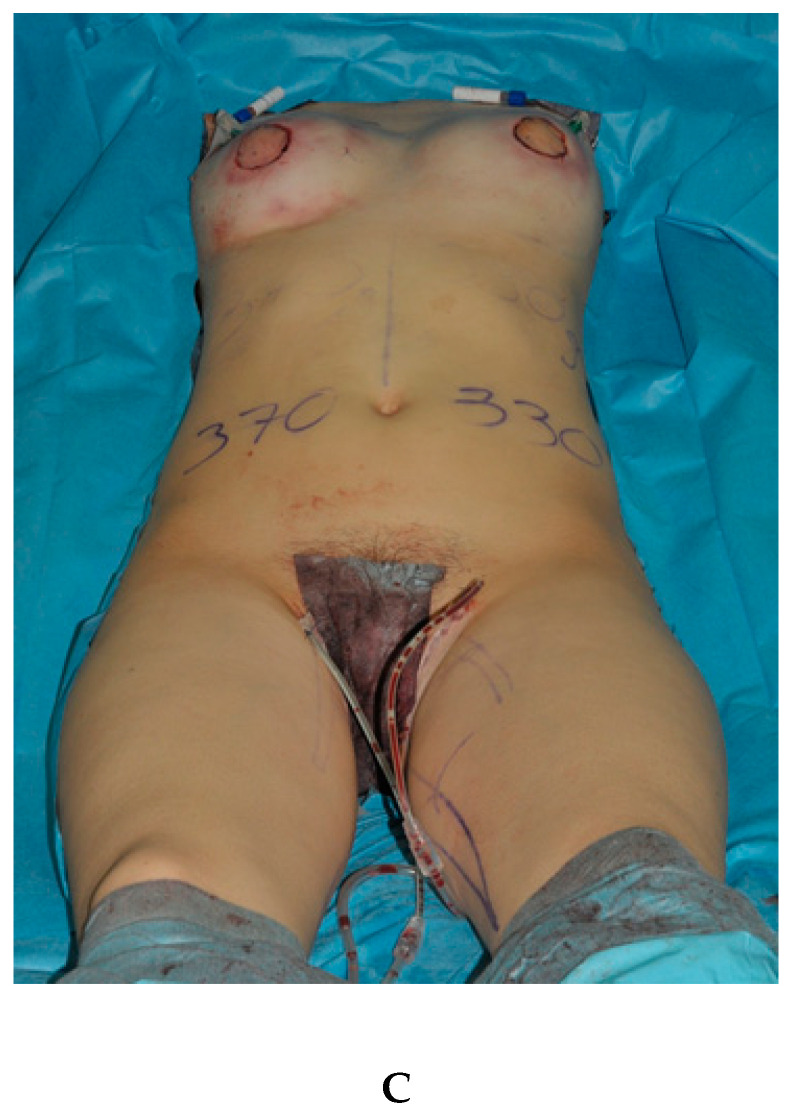
(**A**). Bilateral simultaneous breast reconstruction with TMG flaps. (**B**). Raised bilateral TMG flaps. (**C**). Intraoperative result of a bilateral simultaneous breast reconstruction with TMG flaps.

According to the most suitable donor site for each individual patient, either double DIEP flaps or double TMG flaps were chosen as the reconstructive method (Figure 1). All surgeries were conducted in a standardized two team approach, offering bilateral flap harvest together with simultaneous dissection of the recipient vessels and removal of breast tissue if indicated. The microvascular anastomosis of flap pedicle and recipient vessels was always performed under the microscope using the GEM Synovis Microvascular Anastomotic coupler device^®^ for the venous and single interrupted sutures with 8.0 or 9.0 monofil non-resorbable nylon sutures for the arterial anastomosis. Postoperative protocols were also standardized as the same in each cohort and center. Both senior authors (Gottfried Wechselberger and Thomas Schoeller) have implemented identical pre-, intra- and postoperative protocols in each respective unit, thus, offering a large group of patients without compromising the dataset. Surgical steps for double DIEP reconstructions are shown in Figure 2A–C, and for double TMG reconstructions, in Figure 3A–C.

## 3. Statistics

Data were checked for consistency in terms of typing errors, and ranges were inspected for validity. Crosstabulation tables were analyzed using Pearson’s Chi-squared statistical analysis and Fisher’s exact t-test. Independent Student’s t-tests were used to test means and corresponding 95% CI for difference of means were computed. Levene’s test was used to check for homogeneity of the groups. Relative risks for complication events were calculated. All statistical analyses in this report were performed by use of Social Science Statistics (www.socscistatistics.com).

## 4. Results

Patient age and BMI are shown in Table 1. The average age of patients at the time of surgery was 53 years (standard deviation (SD) 10.0). BMI values of the groups differed significantly, due to the fact that in more adipose patients, a DIEP was more likely considered compared to slim patients, where a TMG flap was considered for reconstruction. The average BMI of the double DIEP group was 28.8 and the average BMI of the double TMG group was 22.2. Patients were followed up on average for 15.6 months (SD 11.57).

In 96% of all cases, patients either currently suffered from, or had a history of, a malignant breast disease in the past. A total of 36% of all cases had a confirmed genetic predisposition for breast cancer development (BRCA gene). In total, 28% (68/238) of operations were primary (immediate) reconstructions, 46% (110/238) secondary and 25% (60/238) were tertiary cases. Impact of comorbidities demonstrated no significant relation to any specific comorbidity. Operating times (cut to suture time) were compared and listed in Table 2.

The overall cut-to-suture time was 7 h and 54 min. No significant difference in operating time could be demonstrated. The cut-to-suture time in the double DIEP group was 7 h and 56 min. The cut-to-suture time in the double TMG group was slightly shorter with 7 h and 49 min. The overall postoperative revision rate was 30.25%. In total, 25% of double DIEP patients needed a postoperative revision compared to 39.5 % of double TMG patients. No significant difference could be found statistically (*p* = 0.0973). Overall complication rate, including all minor and major complications, ranging from the minor wound dehiscence to total flap loss, treated either operatively or conservatively, was 52.1%. Double DIEP patients had significantly fewer overall complications (44.7% vs. 65.1 %, *p* = 0.0325).

**Figure 4:**
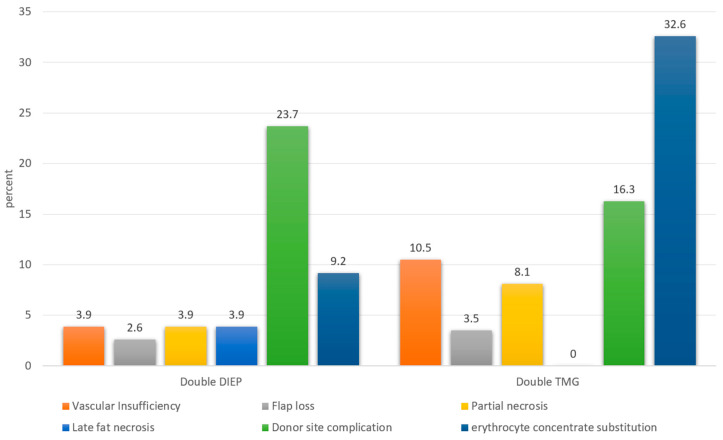
Categorized complications in the double DIEP and double TMG group.

Complications are listed in detail in Table 3 and Figure 4. Vascular insufficiency was observed in 6.3% of all flaps. In double TMG flaps, a vascular insufficiency (e.g., arterial or venous thrombosis of the pedicle) was observed in 10.5%, compared to 3.9% in double DIEP flaps (*p* = 0.0468). Relative risk (RR) was calculated to be 2.65 (RR vascular insufficiency, double TMG vs. double DIEP). Partial flap necrosis was observed in in 5.5% of flaps. In total, 8.1% of double TMG flaps vs. 3.9 % of double DIEP flaps suffered partial necrosis postoperatively, which was not significant (*p* = 0.1716, RR 2.06). 3.5% of double TMG and 2.6% of double DIEP flaps were not salvageable (*p* = 0.7071, RR 1.33). Late stage fat necrosis was observed in 3.9 % of double DIEP cases compared to 0% of double TMG cases (*p* = 0.0898).

Donor site complications were observed in 21% of all patients. In total, 23.7% of double DIEP patients had donor site complications compared to 16.3% of double TMG patients (*p* = 0.9075, RR 1.45).

**Figure 5:**
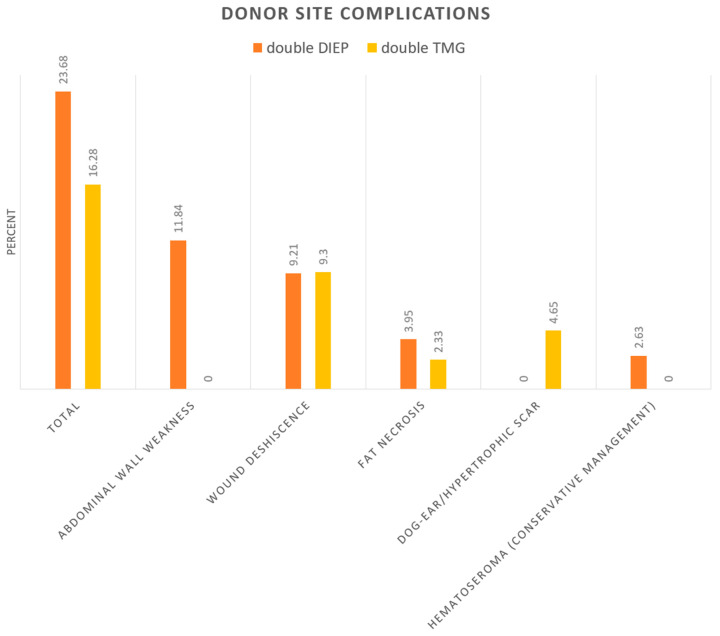
Donor site morbidity in detail.

Of all double TMG patients, 32.6% required postoperative erythrocyte concentrate substitution compared to 9.2% in the double DIEP group (*p* = 0.0013, RR 3.53). The need for postoperative lipofilling was significantly higher in double TMG patients (65.1% vs. 38.2 %, *p* = 0.0047, RR 1.71). The rates for general postoperative minor surgical corrections did not significantly differ between both groups. Double DIEP patients had an average of 1.24 postoperative corrections, compared to 0.98 of double TMG patients (*p* = 0.3209). Overall, 26.1% (31/119) patients with complications had a positive smoking history. Smoking history showed no significant correlation with complications. However, an 11% higher rate of donor site complications was observed in smokers (29.03% vs. 18.18%, *p* = 0.2022, RR 1.6). Donor site complications were classified as minor or major in terms of their severity (Table 4, Figure 5).

## 5. Discussion

Simultaneous bilateral breast reconstruction with autologous tissue remains a very complex surgery, with enormous short-term peri- and postoperative stress for the patient and surgeon.

The risk–benefit ratio of bilateral simultaneous breast reconstructions is still under debate. There are up to six times higher complication rates bi- compared to unilateral breast reconstructions with free flaps and a two times higher risk for vascular insufficiency, and need for a revision of the anastomosis were reported [12]. Additionally, the risk for major adverse events (severe general operation risks, pneumonia, blood transfusions, longer postoperative intubation period, etc.,) peri- and postoperatively increases enormously after operations with a duration longer than 6 h [13].

Nevertheless, simultaneous autologous breast reconstruction represents the most favorable reconstructive option after a bilateral mastectomy for a number of reasons.

In primary (immediate) bilateral breast reconstructions, the psychological stress associated with the mastectomy operation itself can be eliminated or at least reduced to a minimum, since patients do not have to go through the trauma of a missing breast. In delayed (secondary or tertiary) cases, a simultaneous bilateral breast reconstruction likewise offers a significant reduction in surgeries, since both breasts can be reconstructed in one single operation. The simultaneous approach therefore limits surgical trauma, consecutive anesthesia and multiple hospital stays, consequently being more cost efficient. Another advantage is that breast symmetry is easier achieved when the same procedure is simultaneously performed bilaterally [14].

The majority of previous studies compared uni- and bilateral breast reconstructions using DIEP flaps [15,16] or evaluated a single flap type for breast reconstruction, such as superior gluteal artery perforator (SGAP) flaps [17,18] or TMG flaps [6]. A cohort study comparing two large patient cohorts that underwent bilateral simultaneous reconstructions with either double DIEP or double TMG flaps has not been conducted so far. To our knowledge, the largest series of bilateral breast reconstructions was published by the senior author of this study [19], where 26 bilateral breast reconstructions were retrospectively analyzed. A number of smaller studies were conducted by Fansa et al. [20], Buntic et al. (12 bilateral cases each) [21], Vega et al. (six bilateral cases) [22] and Fattah et al. (five bilateral cases) [23]. However, in all studies except the recent study by Bodin et al. (seven bilateral cases) [6], surgical evaluation did not differentiate unilateral and bilateral cases.

No significant difference in operating time could be observed comparing both flaps. Average cut-to-suture time was 7 h and 54 min for all procedures combined. The double TMG procedure only finished approximately 7 min quicker (7 h 49 min) compared to the double DIEP procedure (7 h 56 min) (*p* = 0.698). 

A high overall complication rate of 52.1% for all procedures was observed in the present study. Double DIEP patients had a significantly lower overall complication rate (44.7% vs. 65.1 %, *p* = 0.0325). The high percentages reflect the complexity of the surgery on the one hand, but also the rigorous recording of our data on the other hand. The majority of recorded complications were minor e.g., uncomplicated wound dehiscences or minimally hypertrophic scars which did not need surgical revision. The overall postoperative surgical revision rate was relatively high with 30.25%, including vascular insufficiency, partial and total flap necrosis as well as hemato-seromas, or early postoperative wound breakdown.

Double TMG flaps had a higher rate of vascular insufficiencies (10.5 % vs. 3.9 %, *p* = 0.0468, RR 2.65), which is supposed to be due to the smaller vessel caliber and shorter pedicle length. Thus, the microsurgical procedure is more complex. The higher rate of erythrocyte concentrate substitution is supposed to be a logical consequence of a higher rate of vascular problems and the following revisions needed.

The risk for arterial and venous thrombosis and complete flap failure in both groups were comparable to general literature observation [21,22,23,24,25]. A generally higher risk for flap failure in bilateral compared to unilateral reconstructions is still under debate. While the risk for flap failure is estimated around 1–5% in unilateral reconstructions [26], bilateral reconstructions in the immediate and delayed setting demonstrated a similar flap failure rate in the present study, with 3.5% in double TMG reconstructions, and 2.6% in double DIEP reconstructions, respectively (*p* = 0.7071, RR 1.33). A slightly higher rate of flap failure among bilateral reconstructions with double DIEP flaps is believed to be a consequence of the obligation to anastomose both pedicles of the abdominal flap compared to unilateral reconstruction, in which the ideal pedicle and perforator system may be chosen, as previously proposed by Rao et al. [25].

The double TMG flap demonstrated a decreased donor site morbidity compared to the double DIEP (23.7% (double DIEP) vs. 16.3 % (double TMG), *p* = 0.9075, RR 1.45). A less severe donor site morbidity, mainly including postoperative hemato-seromas and wound break down in the double TMG flap group was observed. Meanwhile, 11.8% of all double DIEP cases suffered a postoperative abdominal wall weakness or a postoperative hernia, which represents a major donor site morbidity that is difficult to surgically address in a revision.

The need for postoperative lipofilling was significantly higher in double TMG patients (65.1% vs. 38.2%, *p* = 0.0047, RR 1.71) which was expected due to decreased flap size compared to the double DIEP flap, and therefore, more occasionally, volume insufficiencies. In a recently published study by Park et al., fat grafting was needed in 13 of 22 (59%) TMG patients after unilateral breast reconstruction [27]. Since a majority of patients will undergo postoperative symmetrization procedures or a nipple areola complex reconstruction, the need for postoperative lipofilling represents no limitation to use double TMG flaps for reconstruction.

Concerning evaluation of the overall aesthetic outcome of these procedures, further prospective studies are needed. Although the TMG is based on muscle, donor site morbidity is low, which is also shown in the present study. Patients have no functional problems after surgery and the scar is hidden in a naturally concealed fold. Conclusively, this flap is an ideal option for young women with small to medium sized breasts and failure of excess abdominal tissue. For older women, who would profit from abdominoplasty anyway, the DIEP procedure might be more favorable. Regarding especially the elderly population, another advantage of the double DIEP might be the easier postoperative mobilization compared to the TMG patients. According to the authors’ experience, both flaps have the potential to offer excellent aesthetic results if used for the right indication. 

The impact of comorbidities and general patient risk factors (smoking, diabetes, peripheral vascular disease, etc.,) on the study has major limitations due to a general selection bias of both patient cohorts. Plastic surgeons might consider only relative healthy people for these procedures. The only conspicuous value was a non-significant 11% higher rate of donor site complications for smokers in general (29.03% vs. 18.18%, *p* = 0.2022, RR 1.6). Another limitation might be the retrospective character of the study and the slightly different sample sizes of the two groups.

## 6. Conclusions

Simultaneous bilateral TMG and DIEP breast reconstruction has a comparable operating time. Complication analysis favors the double DIEP procedure, with one exception: The donor site morbidity, which is lower and less severe in the double TMG procedure. Later fat grafting was more frequently needed after TMG reconstructions. Further studies, preferably of prospective nature, are needed to evaluate the benefit of bilateral simultaneous breast reconstructions.

## Figures and Tables

**Table 1:** Patient age and BMI of study groups.

Table 1	Total	Double DIEP	Double TMG
	*n* = 119	*n* = 76	*n* = 43
Age-years			
mean	53	54.0	52.4
standard deviation	10	9.3	11.2
BMI kg/m²			
mean	26.4	28.8	22.2
standard deviation	5.5	5.0	3.6

**Table 2:** Comparison of cut to suture time (minutes).

Table 2	Total	Double DIEP	Double TMG
Cut to suture time			
mean	473.5	476.4	468.5
standard deviation	106.7	103.4	113.2
*p* = 0.698			

**Table 3:** Comparison of complications and Relative Risk calculation.

Table 3	Total	Double DIEP	Double TMG	*p*-Value
vascular insufficiency	15/238 (6.3 %)	6/152 (3.9 %)	9/86 (10.5 %)	0.0468
Relative Risk double TMG vs. double DIEP:	2.65			
partial flap necrosis	13/238 (5.5 %)	6/152 (3.9 %)	7/86 (8.1 %)	0.1716
Relative Risk double TMG vs. double DIEP:	2.06			
flap loss	7/238 (2.9 %)	4/152 (2.6 %)	3/86 (3.5 %)	0.7071
Relative Risk double TMG vs. double DIEP:	1.33			
late fat necrosis	6/238 (2.5 %)	6/152 (3.9 %)	0/86 (0.0 %)	0.0898
Relative Risk double TMG vs. double DIEP:	0.00			
donor site complication	25/119 (21.0 %)	18/76 (23.7 %)	7/43 (16.3 %)	0.9075
Relative Risk double DIEP vs. double TMG:	1.45			
erythrocyte concentrate substitution	21/119 (17.6 %)	7/76 (9.2 %)	14/43 (32.6 %)	0.0013
Relative Risk double TMG vs. double DIEP:	3.53			

**Table 4:** Donor site morbidity in detail.

Table 4	Double DIEP (%)	Double TMG (%)
Total	23.68	16.28
abdominal wall weakness/Hernias	11.84	0
wound deshiscence	9.21	9.3
fat necrosis	3.95	2.33
dog-ear/hypertrophic scar	0	4.65
Hematoseroma (conservative management)	2.63	0
